# Guideline compliance for bridging anticoagulation use in vitamin-K antagonist patients; practice variation and factors associated with non-compliance

**DOI:** 10.1186/s12959-019-0204-x

**Published:** 2019-08-05

**Authors:** M. J. Moesker, J. F. de Groot, N. L. Damen, N. R. Bijsterveld, J. W. R. Twisk, M. V. Huisman, M. C. de Bruijne, C. Wagner

**Affiliations:** 1 Department of Public and Occupational Health, Amsterdam Public Health Research Institute, Amsterdam UMC, Vrije Universiteit Amsterdam, De Boelelaan 1117, 1081BT Amsterdam, The Netherlands; 2 Netherlands Institute for Health Services Research (NIVEL), Utrecht, The Netherlands; 3 Department of Quality and Safety, Elizabeth Tweesteden Ziekenhuis, Tilburg, The Netherlands; 4 Department of Cardiology, Flevoziekenhuis, Almere, The Netherlands; 5 Department of Epidemiology and Biostatistics, Amsterdam UMC, Vrije Universiteit Amsterdam, Amsterdam, The Netherlands; 6 Department of Thrombosis and Hemostasis, Leiden University Medical Centre, Leiden, The Netherlands

**Keywords:** Anticoagulants, Perioperative care, Practice guideline, Quality of healthcare, Coumarins

## Abstract

**Background:**

Bridging anticoagulation is used in vitamin-K antagonist (VKA) patients undergoing invasive procedures and involves complex risk assessment in order to prevent thromboembolic and bleeding outcomes.

**Objectives:**

Our aim was to assess guideline compliance and identify factors associated with bridging and especially, non-compliant bridging.

**Methods:**

A retrospective review of 256 patient records in 13 Dutch hospitals was performed. Demographic, clinical, surgical and care delivery characteristics were collected. Compliance to the American College of Chest Physicians ninth edition guideline (AT9) was assessed. Multilevel regression models were built to explain bridging use and predict non-compliance.

**Results:**

Bridging use varied from 15.0 to 83.3% (mean = 41.8%) of patients per hospital, whereas guideline compliance varied from 20.0 to 88.2% (mean = 68.5%) per hospital. Both established thromboembolic risk factors and characteristics outside thromboembolic risk assessment were associated with bridging use. Predictors for overuse were gastrointestinal surgery (OR 14.85, 95% CI 2.69–81.99), vascular surgery (OR 13.01, 95% CI 1.83–92.30), non-elective surgery (OR 8.67, 95% CI 1.67–45.14), lowest 25th percentile socioeconomic status (OR 0.33, 95% CI 0.11–1.02) and use of VKA reversal agents (OR 0.22, 95% CI 0.04–1.16).

**Conclusion:**

Bridging anticoagulation practice was not compliant with the AT9 in 31.5% of patients. The aggregated AT9 thromboembolic risk was inferior to individual thromboembolic risk factors and other characteristics in explaining bridging use. Therefor the AT9 risk seems less important for the decision making in everyday practice. Additionally, a heterogeneous implementation of the guideline between hospitals was found. Further research and interventions are needed to improve bridging anticoagulation practice in VKA patients.

**Electronic supplementary material:**

The online version of this article (10.1186/s12959-019-0204-x) contains supplementary material, which is available to authorized users.

## Background

Long-term use of oral anticoagulants such as vitamin-K antagonists (VKA) reduces the risk of thromboembolic events in patients with atrial fibrillation, venous thromboembolism or mechanical heart valves [1–3]. When these patients undergo invasive procedures, such as surgery, the anticoagulant therapy often needs interruption to reduce bleeding. This interruption can increase the risk of thromboembolic complications [4]. In an effort to reduce this risk, short-acting low molecular weight heparin (LMWH) or unfractionated heparin (UFH) are temporarily administered. This is known as ‘bridging anticoagulation’ [5–7].

In general, anticoagulants are consistently identified in adverse event studies as factors involved in preventable adverse events [8, 9], partially occurring in the context of bridging [10].

Due to the risks involved, bridging anticoagulation urges a careful trade-off between thromboembolic and bleeding risk [11, 12]. Consequently, clinicians are required to perform a thorough risk assessment as part of the decision-making in perioperative VKA management.

The American College of Chest Physicians’ *Antithrombotic Therapy and Prevention of Thrombosis, Ninth Edition* guideline (AT9) published in 2012 includes recommendations for this risk assessment by classifying patients in low, moderate or high thromboembolic risk [4]. Bridging is only explicitly recommended for high-risk patients, but might be considered for moderate risk patients too based on individual patient and surgical factors.

**Fig. 1:**
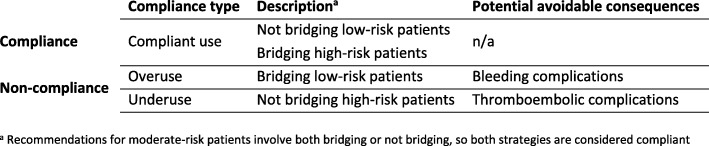
A typology of guideline compliance in perioperative VKA management based on the American College of Chest Physicians’ *Antithrombotic Therapy and Prevention of Thrombosis, Ninth Edition guideline*

Compliance to the AT9 risk stratification and similar guidelines related to bridging is suboptimal [13–15]. Non-compliant bridging can be differentiated in underuse or overuse of bridging anticoagulation. Underuse refers to withholding bridging anticoagulation in high thromboembolic risk patients and overuse refers to unnecessarily administering bridging anticoagulation in low thromboembolic risk patients (Fig. 1). Underuse exposes patients to a higher risk of thromboembolic complications whereas overuse exposes patients to a higher risk for bleeding complications [13–16].

Both bleeding and thromboembolic complications can have serious consequences for patients’ mortality and morbidity [3, 17]. Keeping non-compliant bridging strategies at a minimum should therefore be pursued. Which patients are at risk for non-compliant bridging strategies is relatively unknown. Together with the risks involved around non-compliant bridging, and accumulating evidence reporting up to a 5-fold increased bleeding incidence when bridging is used, identifying patients at risk for a non-compliant bridging strategy is important in reducing preventable mortality and morbidity [11, 12, 18].

Therefore, this study aims to determine guideline compliance of bridging anticoagulation in everyday practice and identify factors associated with bridging use, especially predictors for non-compliant under- and overuse of bridging anticoagulation in Dutch hospitals.

## Methods

### Study design and population

**Fig. 2:**
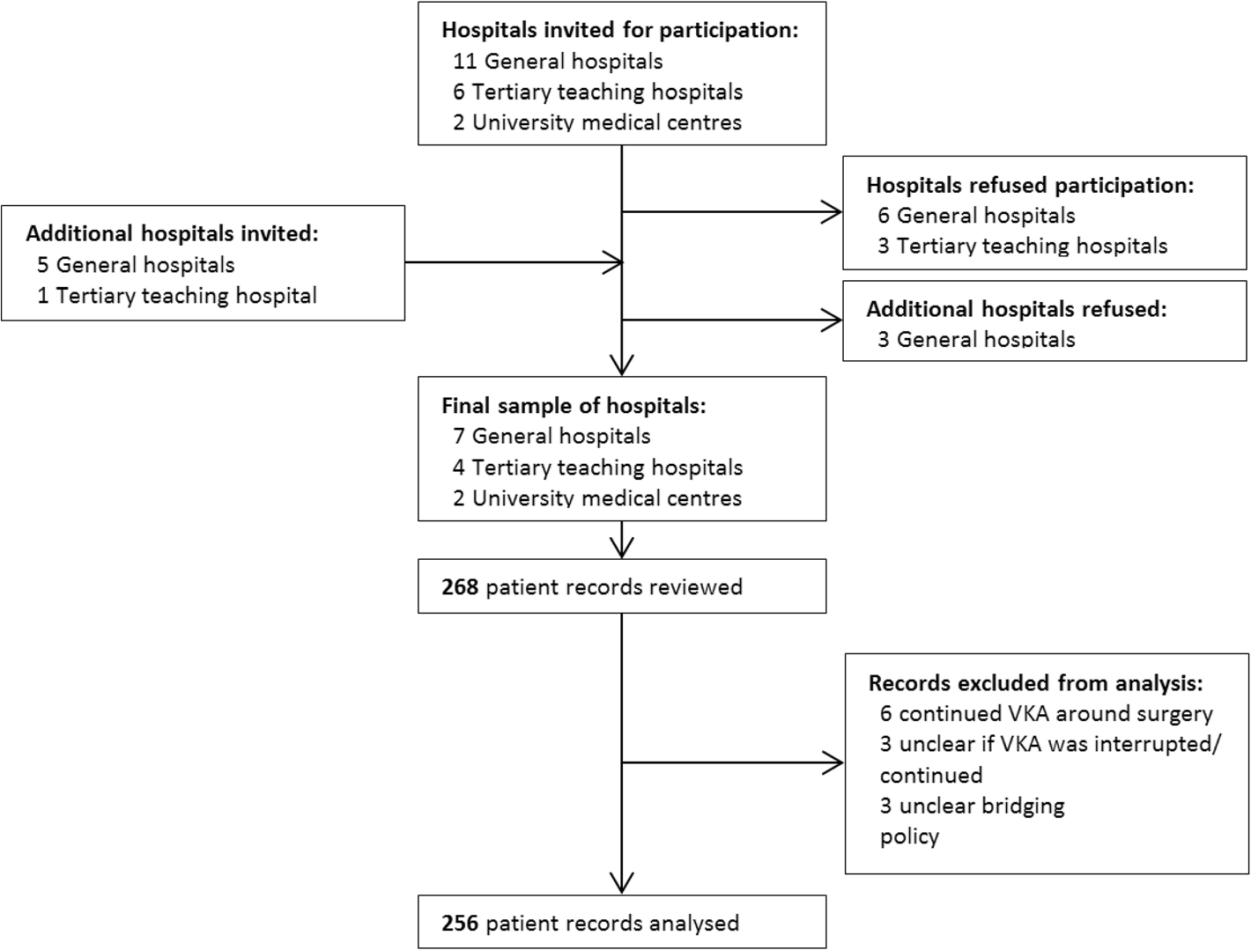
Hospital sample and patient record flowchart

Our current study is part of a larger study evaluating the quality of anticoagulant management in Dutch hospitals by retrospectively reviewing patient records [19]. The hospital sample was stratified by type: university, tertiary teaching, and general hospitals. Within these strata a random selection of hospitals was made while accounting for a proper representation of urban and rural based hospitals. In total, 25 hospitals were invited in two waves of which 13 hospitals participated including two university, four tertiary teaching and seven general hospitals (Fig. 2). Twenty records of patients on long-term VKA, admitted in three consecutive months between June to December 2015 were randomly selected for reviewing the bridging anticoagulation policy. Randomisation of eligible patient records was executed by hospital or research personnel using a random number generator available in common spreadsheet applications.

In case of the absence of a required (section of a) health record, a replacement was randomly selected instead. Inclusion criteria were: age ≥ 18 years, length of stay ≥24 h, undergoing acute or elective surgical procedure using general and/or spinal/epidural anaesthesia. Exclusion criteria were: psychiatric or gynaecologic/obstetric ward admission, admission from or discharge to other hospitals, trauma other than hip fractures on admission, pregnancy or six weeks postpartum and palliative care admission. We excluded patients from analysis if the bridging policy was not recorded, preventing the bridging classification or in case of continued VKA during surgery, making bridging unnecessary (Fig. 2).

### Guideline selection

At the time of data collection in 2015 the Dutch guideline that encompassed bridging anticoagulation in VKA patients was the *Guideline for Diagnostics, Prevention and Treatment of Venous Thromboembolism and Secondary Prevention of Arterial Thrombosis* released by the former Dutch Quality Institute for Healthcare (CBO) in 2008 [20]. This guideline however was an adoption of the ACCP guideline for warfarin patients released back in 2004 [21]. During study preparations it became apparent that in 2015, current practice had moved on and the CBO guideline, at least partially, reflected outdated evidence regarding bridging anticoagulation. Especially since the ACCP updated their guidelines in 2008 and 2012. Several hospitals that were included in our study already pointed out that the AT9 recommendations regarding bridging were incorporated in local protocols. Taken altogether, using the AT9 as a frame of reference for the current study was regarded as the most appropriate.

### Patient record review and compliance assessment

The patient record review consisted of two phases. Phase one involved the extraction of all data from patient records. Phase two involved the actual bridging anticoagulation evaluation. A panel of five experts in the thrombosis and haemostasis field, all of whom participated in guideline development on antithrombotic care, were consulted throughout the two phases. The panel contributed in developing standardized case report forms for phase one and classification models for determining guideline compliance in phase two.

#### Phase one: data extraction

In phase one, LMWH and UFH administration data was extracted from the patient records. Other data extracted were: demographic, clinical, surgery, and care delivery characteristics (Additional file 1: Tables S2-S3). For demographic characteristics variables such as age, sex and socioeconomic status (SES) were collected. SES was extracted from open source data available from the Netherlands Institute for Social Research and matched with our data using the patients four-digit zip code [22]. Clinical characteristics primarily included risk factors used for determining the AT9 thromboembolic risk classification [4]. These were supplemented with characteristics used in thrombo-prophylaxis risk assessment [23, 24] and patient related risk factors for bleeding as well as surgical bleeding [25, 26]. A previous bleeding event was defined as any bleeding coming to the attention of the treating physician.

In absence of an alternative validated instrument, determination of surgical bleeding risk was based on a Dutch expert consensus classification of procedures in low-, medium- or high-risk strata [27]. Other surgical characteristics extracted were: type, duration, whether a second surgery was performed and type of anaesthesia. Lastly, care delivery characteristics based on adverse event studies, such as weekend admission or surgery, were extracted [28–30].

Data extraction took place from January to August 2016. Trained research assistants and one researcher (MM) extracted all patient record data. The study protocol was approved by the medical ethics committee of the VU University Medical Centre, Amsterdam, The Netherlands and the informed consent was waived because of the use of patient record data only (protocol number: 2015/430).

#### Phase 2: classification of guideline compliance

In phase two, patients were classified on thromboembolic risk according to the AT9 (Additional file 1: Table S1) and bridging anticoagulation use. In case of multiple indications for VKA use (e.g. atrial fibrillation and mechanical heart valve), the indication associated with the highest thromboembolic risk was used for determining guideline compliance.

The bridging anticoagulation classification was based on postoperative administration of LMWH or use of continuous intravenous UFH infusion. Prophylactic LMWH regimens were not classified as bridging. See Additional file 1: Table S2 for details on LMWH dosages classified as bridging anticoagulation. Compliance with the guideline was defined as withholding bridging anticoagulation in low thromboembolic risk patients and administering bridging anticoagulation in high thromboembolic risk patients. *Underuse* was defined as not bridging high thromboembolic risk patients. *Overuse* was defined as bridging low thromboembolic risk patients (Fig. 1). For moderate-risk patients, both bridging and non-bridging were defined as compliant, since the AT9 does not recommend a specific approach for this patient group.

### Statistics and model development

To describe the study population regarding demographic, clinical, surgical and care delivery characteristics we used descriptive statistics. Characteristics associated with bridging use were analysed with univariable and multivariable logistic regression. The dependent variable in our first model was: *bridging* versus *no bridging*. Independent variables considered for entry in the model were the aforementioned demographic, clinical, surgical and care delivery characteristics.

To predict a guideline discordant bridging decision in relation to the AT9 guideline, we created two separate models. One to identify predictors for *overuse* in the low thromboembolic risk population and one to identify predictors for *underuse* in the high thromboembolic risk population. Independent variables in this second and third model were slightly different compared to the first model. We excluded variables for which the exposure to the independent variable did not precede the measurement of the dependent variable (admission on critical or cardiac care unit, length of stay, presence of central venous or spinal and epidural catheters, second surgery performed, laboratory tests during admission). Furthermore, the AT9 thromboembolic risk was not considered as an independent variable since thromboembolic risk served as the foundation for the classification of, under- and overuse of bridging, therefore not being informative.

Univariable logistic regression results are presented as odds ratios (OR) and 95% confidence intervals (95% CI). Results were considered significant if the 95% CI did not intersect unity. Following the univariable analyses, a *p*-value entry level set at < 0.10 was used for a multivariable forward selection procedure. The maximum number of independent variables allowed in the models was based on the 10:1 rule to prevent overfitting [31]. Cases with missing values for independent variables were excluded from the regression analyses. Furthermore, variables with more than 10% missing values were not considered for multivariable modelling.

To enable our models to estimate predictor coefficients independent of possible practice variation between hospitals, we applied a multilevel approach in all regression analyses. Because the patient data were clustered within hospitals a random intercept on hospital level was allowed. C-statistics were calculated to evaluate the discriminative power of the models. A c-statistic of 0.5 to 0.7 is interpreted as a low discriminative power, 0.7–0.9 as moderate and > 0.9 as high. Statistical analyses were performed with SPSS version 22 (IBM, Chicago, IL).

## Results

### Study population

In total, 268 records were reviewed of which 256 records were eligible for bridging anticoagulation analyses (Fig. 2). The mean age of patients was 74.8 (SD = 10.6) years, 55.9% were male. Other characteristics are displayed in Table 1. Atrial fibrillation was the most common indication for VKA use with (74.2%). Thromboembolic risk was low, moderate or high in 52.7, 14.8 and 15.6% of patients respectively. 33 (12.9%) patients used VKA for other indications than AT9 provides recommendations and could thus not be classified according to AT9 thromboembolic risk. In 10 (3.9%) patients the records provided insufficient information for thromboembolic risk classification.

**Table 1:** Demographic, clinical and surgical characteristics for the overall population

	Patients *N* = 256
Demographic characteristics	
Male sex	143 (55.9)
Age (years), mean (SD)	74.76 (10.59)
Clinical characteristics	
AT9 Thromboembolic risk	
Low	135 (52.7)
Moderate	38 (14.8)
High	40 (15.6)
Other VKA indication ^a^	33 (12.9)
Risk factors unknown	10 (3.9)
Atrial fibrillation	190 (74.2)
Mechanical heart valve	20 (7.8)
Venous thromboembolism	34 (13.3)
Previous thromboembolic event during VKA interruption	3 (1.2)
iCVA/TIA	37 (14.5)
Thrombophilia	7 (2.7)
Coronary heart disease	74 (28.9)
Heart failure	20 (7.8)
Hypertension	129 (50.4)
Diabetes mellitus	62 (24.2)
Active cancer/malignancy	54 (21.5)
Previous bleeding^b^	13 (5.1)
VKA regimen	
Acenocoumarol	203 (79.3)
Phenprocoumon	53 (20.7)
Length of stay (days): median (IQR)	6 (3–10)
Surgery characteristics	
Elective	181 (70.7)
Type of 1st surgery	
Urologic	40 (15.6)
Orthopaedic	89 (34.8)
Gastrointestinal	52 (20.3)
Vascular	36 (14.1)
Other	39 (15.2)
Surgical bleeding risk	
High	209 (81.6)
Moderate	44 (17.2)
Low	3 (1.2)

Results are expressed as n (%) unless stated otherwise

*AT9* Antithrombotic Therapy and Prevention of Thrombosis, Ninth Edition guideline, *iCVA* ischaemic cerebrovascular accident, *IQR* inter quartile range, *SD* standard deviation, *TIA* transient ischaemic attack, *VKA* vitamin-K antagonist

^a^ No AT9 risk classification is available for VKA indications other than atrial fibrillation, mechanical heart valves and venous thromboembolism

^b^ Any previous bleeding event annotated in the medical record

Table 2 displays the AT9 thromboembolic risk of the patients for each of the indications for VKA use. The most prevalent thromboembolic risk category was low for atrial fibrillation patients (69%), moderate for venous thromboembolism patients (65%) and high for mechanical heart valve patients (45%).

**Table 2:** AT9 thromboembolic risk for each of the VKA indication groups

	Indication group: n (column %) ^a^		
AT9 Thromboembolic risk	Atrial fibrillation	Mechanical heart valve	Venous thromboembolism
Low	131 (69)	1 (5)	6 (18)
Moderate	19 (10)	4 (20)	22 (65)
High	35 (18)	9 (45)	4 (12)
Unknown ^b^	5 (3)	6 (30)	2 (6)

AT9: Antithrombotic Therapy and Prevention of Thrombosis, Ninth Edition guideline; VKA: vitamin-K antagonist

^a^ Multiple indications are possible

^b^ Insufficient documentation of risk factors in the records, so the AT9 risk could not be determined

### Bridging use and guideline compliance

**Fig. 3:**
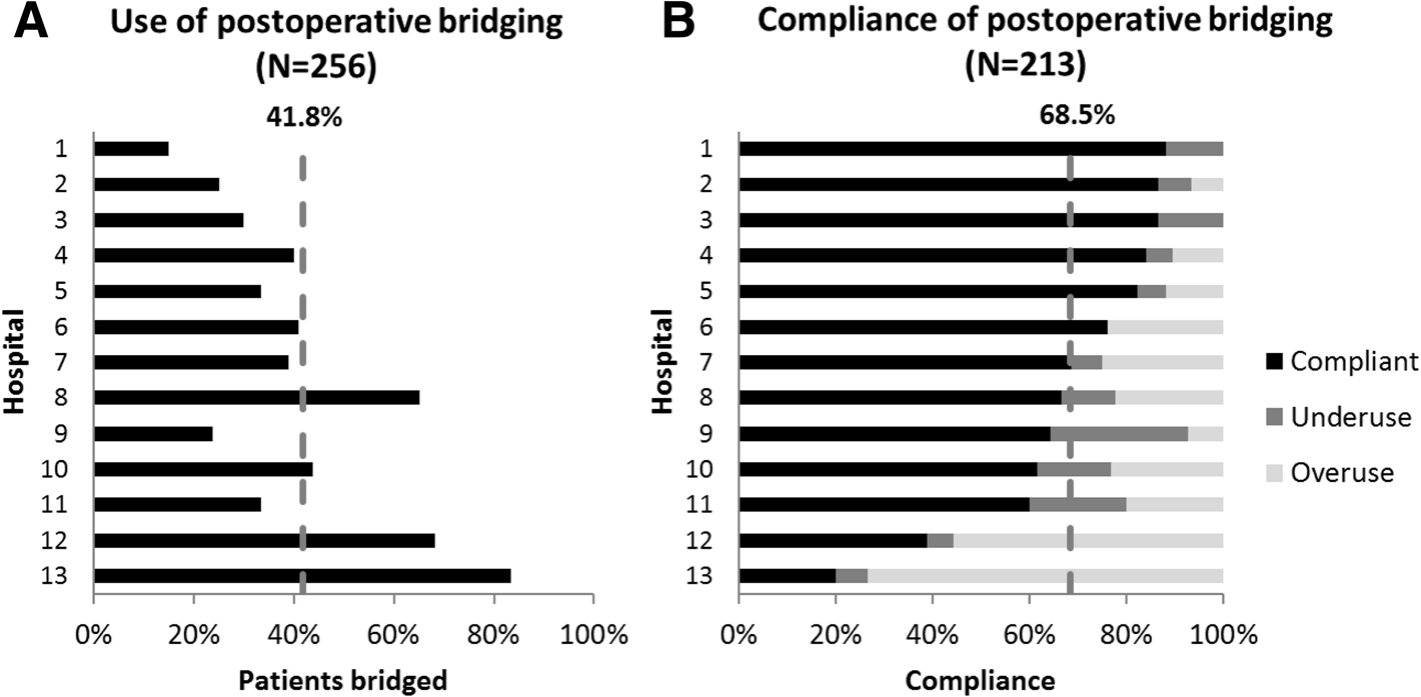
Barcharts displaying the use (**a**) and compliance (**b**) of postoperative bridging anticoagulation per hospital and on average. The dashed vertical lines represent the average

In 107 (41.8%) patients, bridging anticoagulation was used. Bridging rates between hospitals ranged from 15 to 83% of all patients per hospital (Fig. 3a). Based on the AT9 thromboembolic risk recommendations, the decision to apply or withhold bridging anticoagulation was compliant with the guideline in 68.5% of all patients for which the thromboembolic risk could be determined (*N* = 213). Compliance rates for each AT9 risk and VKA indication strata are given in Table 3. Compliance was lowest for high risk atrial fibrillation patients (46%, *N* = 35), low risk venous thromboembolism (50%, *N* = 6) and low risk mechanical heart valve patients (0%, *N* = 1), however the latter two were very small strata. Low risk atrial fibrillation patients on the other hand, comprised the largest stratum in our study (51.2% of the total population), with a compliance of 67%.

**Table 3:** Compliance of postoperative bridging per indication and AT9 thromboembolic risk group

	Compliance of postoperative bridging per indication group: n(%) ^a^		
AT9 Thromboembolic risk	Atrial fibrillation	Mechanical heart valve	Venous thromboembolism
Low	88 (67)	0 (0)	3 (50)
Moderate	19 (100)	4 (100)	22 (100)
High	16 (46)	5 (56)	3 (75)

AT9: Antithrombotic Therapy and Prevention of Thrombosis, Ninth Edition guideline; VKA: vitamin-K antagonist

^a^ Multiple indications are possible

Comparing hospitals, the compliance rate ranged from 20 to 88% of all patients per hospital (Fig. 3b).

### Factors associated with use of bridging anticoagulation

Univariable logistic regression results for the application of bridging are presented in Additional file 1: Table S3. Compared to low-risk patients, moderate thromboembolic risk patients had a significant increased odds (OR 3.36, 95% CI 1.52–7.41) and high-risk patients a borderline insignificant increased odds (OR 2.05, 95% CI 0.95–4.21) for receiving bridging anticoagulation. Furthermore, all three main indications for VKA use were significantly associated with bridging: mechanical heart valve (OR 3.69, 95% CI 1.34–10.20) and venous thromboembolism (OR 2.35, 95% CI 1.09–5.07) patients were more likely to receive bridging anticoagulation while atrial fibrillation patients (OR 0.50, 95% CI 0.27–0.92) were less likely to be bridged.

Characteristics outside the AT9 thromboembolic risk assessment associated with bridging were length of hospital stay (OR 1.07 per day, 95% CI 1.03–1.11), critical or cardiac care unit admission (OR 3.80, 95% CI 1.80–8.05), second surgery (OR 6.45, 95% CI 1.96–21.21), and admission to a university hospital (OR 3.94, 95% CI 1.16–13.35). Lastly, gastrointestinal (OR 3.83, 95% CI 1.50–9.74), vascular (OR 3.74, 95% CI 1.36–10.29) and other (OR 3.06, 95% CI 1.12–8.39) surgery types were positively associated with bridging.

Our multivariable logistic regression analysis included 249 patients and resulted in a model with critical or cardiac care unit admission, second surgery, mechanical heart valve, surgery type, venous thromboembolism, ischeamic CVA or TIA and previous bleeding, as explanatory variables for bridging use. Regression parameters are displayed in Table 4. The model’s power to discriminate between bridged and non-bridged patients was moderate (c-statistic 0.85, 95% CI 0.80–0.90).

**Table 4:** Multivariable logistic regression models for bridging use and overuse of bridging, adjusted for clustering at hospital level

	OR (95% CI)^a^
Model 1, All patients	
Bridging used (reference: no bridging used)	
ICU/CCU stay during admission	4.45 (1.72–11.51)
Second surgery performed	3.21 (0.83–12.49)
Mechanical heart valve	8.10 (2.38–27.50)
Type of 1st surgery (reference category: urologic)	
Orthopaedic	1.10 (0.42–2.91)
Gastrointestinal	3.45 (1.21–9.87)
Vascular	3.21 (1.01–10.21)
Other	3.57 (1.14–11.21)
Venous thromboembolism	3.91 (1.57–9.74)
iCVA/TIA	2.49 (1.02–6.11)
Previous bleeding^b^	3.59 (0.80–16.17)
Model 2, Low TE risk patients:	
Overuse of bridging (reference: compliant use)	
Type of 1st surgery (reference category: urologic)	
Orthopaedic	3.18 (0.60–16.71)
Gastrointestinal	14.85 (2.69–81.99)
Vascular	13.01 (1.83–92.30)
Other	57.30 (5.27–623.62)
Non-elective surgery	8.67 (1.67–45.14)
Lowest 25th percentile SES	0.33 (0.11–1.02)
VKA reversal agent used	0.22 (0.04–1.16)

*CCU* cardiac care unit, *ICU* intensive care unit, *iCVA* ischaemic cerebrovascular accident, *TIA* transient ischaemic attack, *SES* Socioeconomic status, *VKA* Vitamin-K antagonist

^a^ Adjusted for clustering at hospital level

^b^ Any previous bleeding event annotated in the medical record

### Predictors of over- and underuse of bridging anticoagulation

Overuse of bridging anticoagulation occurred in 34.1% of low thromboembolic risk patients and underuse occurred in 52.5% of high thromboembolic risk patients. Univariable logistic regression results for both over- and under use are presented in Additional file 1: Table S4. Within low risk patients, positive associations for overuse of bridging were found for non-elective surgery (OR 2.72, 95% CI 1.03–7.19), gastrointestinal (OR 15.87, 95% CI 3.02–83.42), vascular (OR 9.58, 95% CI 1.49–61.42) and other (OR 27.43, 95% CI 3.49–215.38) surgery types, and admission to a university medical centre (OR 9.01, 95% CI 1.05–77.57).

The high risk patient strata was of limited size (40 patients). Hence, the power to capture a significant association for underuse within this population was limited. Only a borderline insignificant effect for surgery duration was observed (OR 0.98 per minute, 95% CI 0.96–1.00).

The multivariable logistic regression parameters for predicting overuse of bridging are presented in Table 4. Surgery type and non-elective surgery were positive predictors whereas membership of the lowest 25th percentile SES and VKA reversal agent use were negative predictors for overuse of bridging. The discriminative power for predicting overuse of bridging was high (c-statistic 0.91, 95% CI 0.86–0.97).

## Discussion

### Bridging use and guideline compliance

In 31.5% of the patients in our sample the bridging anticoagulation policy was not compliant with the American College of Chest Physicians’ *Antithrombotic Therapy and Prevention of Thrombosis, Ninth Edition* recommendations. Bridging was used during 41.8% of VKA interruptions, lower than reported in existing literature [13, 14, 16]. As a result, the 52.5% underuse of bridging was higher in our study compared with 36.8 and 13.0% reported in other studies [13, 14]. Conversely, the 34.1% overuse of bridging in low risk patients is on the lower side of the spectrum of overuse rates reported by others that ranged between 28.7 and 84.3% [13–15].

However, these low-risk patients represent over 50% of the VKA patient population in our study and are mostly patients with atrial fibrillation. Although the exact number of VKA patients undergoing surgery in the Netherlands is unavailable, there are over 460.000 VKA patients present [32]. Based on our findings, overuse of bridging is likely to occur in a substantial amount.

In light of accumulating evidence towards increased bleeding risk among bridged patients this overuse warrants attention. In a meta-analysis of predominantly observational studies, Siegal et al. 2012 found that bridged patients had a 5-fold increased risk for overall, and a 3-fold increased risk for major bleeding [11, 12]. This was confirmed by Douketis et al. in 2015 in the BRIDGE-trial, where the risk of major bleeding was 0.41; 95% CI 0.20–0.78 for non-bridged patients relative to bridged patients [12]. Bleeding complications occurring in bridged patients have been found to increase the risk for reoperation and prolonged hospitalisation [33, 34]. Moreover, the BRIDGE-trial also found that non-bridging was not associated with an increased incidence of thromboembolic complications, which contradicts the rationale behind bridging anticoagulation.

Given this evidence and our study results, low risk atrial fibrillation patients undergoing surgery comprise a large group of patients who might benefit the most from improvement efforts to reduce bridging overuse and reduce adverse bleeding outcomes.

### Factors associated with bridging anticoagulation, predicting over- and underuse

To understand why current bridging practice is not always in line with guideline recommendations we aimed to identify characteristics associated with bridging use. The associations found for atrial fibrillation, mechanical heart valve and venous thromboembolism patients correspond with the findings of others where most atrial fibrillation patients did not receive bridging and most mechanical heart valve and venous thromboembolism patients were at least at moderate thromboembolic risk justifying bridging anticoagulation use [14, 35, 36]. Regarding, the aggregated AT9 thromboembolic risk strata, the moderate and high risk strata were more likely to receive bridging, which is to be expected. However, the introduction of individual thromboembolic risk factors and other characteristics in our multivariable analysis rendered the association insignificant. Translating this to practice, it can be argued that awareness to the aggregated AT9 thromboembolic risk might be limited to individual risk factors that make up the AT9 risk strata. Also, patient characteristics outside the AT9 thromboembolic risk assessment may be involved in the decision to apply bridging. Our study points to several of these.

First, a history of bleeding showed a positive association with bridging. This seems contradictory, and is difficult to explain. One would expect a more conservative approach to using bridging anticoagulation in patients with signs of a previous bleeding. However, only recently the risks of bridging versus uninterrupted anticoagulation were supported with high quality data. Before this, bridging with fast onset and offset heparins seemed the safest option.

Second, bridging use and overuse occurred more frequently in gastrointestinal, vascular and other surgery types compared with urologic and orthopaedic surgery. Perceived thromboembolic risks relative to the surgical procedures can play a role. The AT9 thromboembolic risk classification does not formally include this but designates certain high thromboembolic risk procedures [4]. Furthermore, heterogeneous practice and preferences between medical specialties related to the studied surgery types might be responsible for our findings. A recent survey study underscores this. Flaker et al. (2016), found different perioperative management strategies between medical specialties [37].

Third, Intensive or cardiac care unit admission and a second surgery were associated with higher bridging rates. We think this is possibly explained by factors related with the severity of the patient’s disease and clinical course that we were unable to correct for, such as the inability to take oral medication. In these cases parenteral heparins are a feasible alternative to oral VKAs.

Regarding bridging overuse specifically, primarily surgical characteristics such as type and urgency were predictive for non-compliant use of bridging. Based on these characteristics, the population at which further investigation and improvement efforts should aim for can be narrowed down. Additionally we found that membership of the lowest 25th percentile socioeconomic status was a significant negative predictor for overuse. Besides socioeconomic status being a well-established determinant for health and access to health services [38, 39], associations with guideline compliance have also been found before [40, 41].

Altogether, our exploratory analyses indicate that current bridging anticoagulation practice is not explained by the ACCP’s thromboembolic risk assessment recommendations alone. Our study therefore confirms the findings of several other studies [14, 15, 42, 43]. Why practice is not in accordance with bridging recommendations is relatively unknown. Whether the other associated clinical and surgical characteristics identified, are the result of a conscious assessment in everyday bridging practice, cannot be concluded based on our results.

### Practice variation

Our results also revealed variation between hospitals. Bridging varied from 15 to 83% of patients, similar to a US study where rates ranged from 10 to 88% [36]. Furthermore, hospitals that bridged more frequently had lower compliance rates and higher overuse rates. Thus, higher bridging rates cannot solely be explained by case-mix differences regarding thromboembolic risk.

More likely, a heterogeneous implementation or embedment of guidelines into local processes and protocols results in variations in practice. For example, differences in responsible professionals in terms of specialty or experience might affect the risk assessment for bridging anticoagulation. The existence of variation like this was endorsed in a Dutch report revealing substantial differences between hospital’s adaptations of an integrated anticoagulant care guideline. This guideline predominantly contains recommendations regarding care processes, responsibilities and communication for anticoagulant care. Among others, a major difference observed was the instalment of dedicated anticoagulation committee’s or case managers while other hospitals were less progressive [44].

### Strengths and limitations

Our study has several strengths and limitations. Our multi-centre design is a strength and informs us on bridging anticoagulation practice in a variety of hospitals while the entire sample was representative for the Dutch hospital distribution. The voluntary hospital participation can be regarded as a limitation that could have introduced selection bias on hospitals’ awareness or priority regarding anticoagulant care. The retrospective approach is another strength in ensuring results not being influenced by carrying out the study but rather reflect everyday care. On the other hand, the dependency of routinely recorded medical data might be a limitation. Although efforts were made to retrieve all required information, some records were found to be too incomplete to include and others were prone to missing information, especially details required for thromboembolic risk classification of mechanical heart valve patients. While this might have introduced some bias to our results, it also stresses the importance of adequate record quality.

Additionally, we wish to nuance non-compliance. First, our study was carried out in a transition period between an outdated guideline and the adoption of the AT9. Second, the reasoning behind informed guideline deviations were not collected from the medical records. Hence, we wish to point out that non-compliance with the guidelines does not necessarily reflect poor care.

Lastly, the limited amount of high risk patients in our sample prevented a multivariable analysis for bridging underuse.

## Conclusions and implications

In 31.5% of the patients the bridging anticoagulation policy was not in line with the AT9 recommendations.

Improvement efforts targeted at low-risk atrial fibrillation patients are expected have the biggest effect on overall compliance and potentially adverse outcomes since these patients represented over 50% of our study population. Bridging was predominantly related with individual clinical and surgical characteristics rather than the aggregated AT9 thromboembolic risk. Overuse of bridging, was the most prevalent form of non-compliance. Gastrointestinal, vascular and non-elective surgery were risk factors for overuse. Underuse of bridging in high-risk patients was less prevalent and no significant risk factors were identified. Our results raise the question whether AT9 risk assessment sufficiently reflects the risks that are perceived in everyday practice or if they are deviant for other reasons. Also a large variation in bridging practice between hospitals was observed, where hospitals with high bridging rates had lower compliance rates and vice versa.

Based on our study, several implications can be thought of to improve bridging anticoagulation practice. 1) Qualitative research can inform us on the reasons and mechanisms leading to differences between everyday practice and what is advocated in the guidelines. 2) The characteristics associated with non-compliant bridging, should be taken in to account in interventions aimed at improving decision making in bridging anticoagulation, e.g. electronic decision support systems. 3) Variation between hospitals regarding the implementation and embedment of guidelines in local practice should be studied to identify factors related with practice variation.

## Additional file

Additional file 1: **Table S1.** Thromboembolic **r**isk stratification used. Based on ACCP 2012 guideline [9]. **Table S2.** LMWH dose thresholds used for bridging anticoagulation classification. **Table S3.** Demographic, clinical, surgical and care delivery characteristics for the overall population and univariable logistic regression results for bridging use, adjusted for clustering on hospital level. **Table S4.** Demographic, clinical, surgical and care delivery characteristics by compliance status for low- and high thromboembolic risk patients. Additionally, univariable logistic regression results for under and overuse of bridging, adjusted for clustering on hospital level, are presented. (DOCX 40 kb)

## Data Availability

The data that support the findings of this study are available from The Netherlands institute for health services research (NIVEL) but restrictions apply to the availability of these data, which were used under license for the current study, and so are not publicly available. Data are however available from the authors upon reasonable request and with permission of NIVEL.

## Abbreviations

AT9: The American College of Chest Physicians’ *Antithrombotic Therapy and Prevention of Thrombosis, Ninth Edition*
LMWH: Low molecular weight heparin
SES: Socioeconomic status
UFH: Unfractionated heparin
VKA: Vitamin-K antagonist
