# Supplementary material for: Guideline compliance for bridging anticoagulation use in vitamin-K antagonist patients; practice variation and factors associated with non-compliance
Source: Thromb J. 2019 Aug 5;17:15. doi: 10.1186/s12959-019-0204-x (PMC6681479; doi:10.1186/s12959-019-0204-x)
Supplement: Supplementary file 1 — Table S1. Thromboembolic risk stratification used. Based on ACCP 2012 guideline [9]. Table S2. LMWH dose thresholds used for bridging anticoagulation classification. Table S3. Demographic, clinical, surgical and care delivery characteristics for the overall population and univariable logistic regression results for bridging use, adjusted for clustering on hospital level. Table S4. Demographic, clinical, surgical and care delivery characteristics by compliance status for low- and high thromboembolic risk patients. Additionally, univariable logistic regression results for under and overuse of bridging, adjusted for clustering on hospital level, are presented. (DOCX 40 kb) [file 12959_2019_204_MOESM1_ESM.docx]

| **Supplementary table 1 Thromboembolic risk stratification used. Based on ACCP 2012 guideline [9]** | | | |
| --- | --- | --- | --- |
|  | | | |
|  | **VKA indication** | | |
| **Risk stratum** | **Mechanical heart valve** | **Atrial fibrillation** | **Venous thromboembolism (VTE)** |
| **High** | **Any mitral valve prosthesis**  **Any caged-ball or tilting disk aortic valve**  **prosthesis**  **iCVA or TIA within 6 months**  **Previous thromboembolic event during**  **VKA interruption** | **CHA_2_DS_2_-VASC score 8 or 9**  **iCVA or TIA within 3 months**  **CHA_2_DS_2_-VASC score <8 with iCVA**  **or TIA >3 months ago**  **Rheumatic valvular heart disease**  **Previous thromboembolic event**  **during VKA interruption** | **VTE within 3 months**  **Severe thrombophilia (e.g. deficiency**  **of protein C or S or anti-thrombin,**  **anti-phospholipid antibodies; multiple**  **abnormalities)**  **Previous thromboembolic event during**  **VKA interruption** |
| **Moderate** | **Bileaflet aortic valve prosthesis and one or more riskfactors:**   - **Atrial fibrillation** - **iCVA or TIA** - **Hypertension** - **Diabetes** - **Heart failure** - **Age >75 years** | **CHA_2_DS_2_-VASC score 5 to 7** | **VTE within the past 3-12 months**  **Recurrent VTE**  **Non-severe thrombophilia (e.g.**  **heterozygous factor V or prothrombin**  **gene mutation)**  **Active cancer** |
| **Low** | **Bileaflet aortic valve without riskfactors** | **CHA_2_DS_2_-VASC score <5** | **VTE > 12 months and no other risk factors** |
| **CHA_2_DS_2_-VASC= Congestive heart failure, hypertension, age ≥75, diabetes, iCVA/TIA, vascular disease;** iCVA: ischaemic cerebrovascular accident; TIA: transient ischaemic attack | | | |

| **Supplementary table 2 LMWH dose thresholds used for bridging anticoagulation classification.** | |
| --- | --- |
|  | |
| **LMWH** | **Bridging anticoagulation dose threshold** |
| **Dalteparin** | **> 5.000 IE/day** |
|  |  |
| **Nadroparin** | **> 5.700 IE/day** |
|  |  |
| **Tinzaparin** | **> 4.500 IE/day** |
|  |  |

| **Supplementary table 3** Demographic, clinical, surgical and care delivery characteristics for the overall population and univariable logistic regression results for bridging use, adjusted for clustering on hospital level | | | | | | | |
| --- | --- | --- | --- | --- | --- | --- | --- |
|  | | | | | | | |
|  | | | | Bridging | | | |
|  | No. of valid cases | Overall  N=256 |  | No  N=149 | Yes  N=107 |  | OR (95% CI) ^a^ |
| **Demographic characteristics** |  |  |  |  |  |  |  |
| Age: n (%) | 256 |  |  |  |  |  |  |
| *≥80 years* |  | 98 (38.3) |  | 57 (38.3) | 41 (38.3) |  | Ref. |
| 60-79 *years* |  | 138 (53.9) |  | 84 (56.4) | 54 (50.5) |  | 0.94 (0.54-1.65) |
| *<60 years* |  | 20 (7.8) |  | 8 (5.4) | 12 (11.2) |  | 2.11 (0.74-5.98) |
| Female sex: n (%) | 256 | 113 (44.1) |  | 66 (44.3) | 47 (43.9) |  | 1.13 (0.66-1.94) |
| Lowest 25^th^ percentile SES: n (%) | 253 | 96 (37.9) |  | 57 (39.0) | 39 (36.4) |  | 0.91 (0.52-1.60) |
| **Clinical characteristics** |  |  |  |  |  |  |  |
| AT9 Thromboembolic risk: n (%) | 246 |  |  |  |  |  |  |
| *Low* |  | 135 (54.9) |  | 89 (61.4) | 46 (45.5) |  | Ref. |
| *Moderate* |  | 38 (15.4) |  | 16 (11.0) | 22 (21.8) |  | **3.36 (1.52-7.41)** |
| *High* |  | 40 (16.3) |  | 21 (14.5) | 19 (18.8) |  | 2.05 (0.95-4.21) |
| *Other VKA indication ^b^* |  | 33 (13.4) |  | 19 (13.1) | 14 (13.9) |  | 1.67 (0.70-3.98) |
| Atrial fibrillation: n (%) | 256 | 190 (74.2) |  | 119 (79.9) | 71 (66.4) |  | **0.50 (0.27-0.92)** |
| Mechanical heart valve: n (%) | 256 | 20 (7.8) |  | 7 (4.7) | 13 (12.1) |  | **3.69 (1.34-10.20)** |
| Venous thromboembolism: n (%) | 256 | 34 (13.3) |  | 14 (9.4) | 20 (18.7) |  | **2.35 (1.09-5.07)** |
| Previous thromboembolic event during VKA interruption: n (%) | 255 | 3 (1.2) |  | 1 (0.7) | 2 (1.9) |  | 1.77 (0.15-21.26) |
| iCVA/TIA: n (%) | 252 | 37 (14.7) |  | 19 (12.9) | 18 (17.1) |  | 1.46 (0.70-3.07) |
| Thrombophilia: n (%) | 256 | 7 (2.7) |  | 1 (0.7) | 6 (5.6) |  | 8.08 (0.91-71.80) |
| Coronary heart disease: n (%) | 256 | 74 (28.9) |  | 43 (28.9) | 31 (29.0) |  | 0.87 (0.48-1.57) |
| Heart failure: n (%) | 252 | 20 (7.9) |  | 11 (7.4) | 9 (8.6) |  | 0.94 (0.36-2.49) |
| Hypertension: n (%) | 256 | 129 (50.4) |  | 76 (51.0) | 53 (49.5) |  | 0.98 (0.58-1.67) |
| COPD: n (%) | 256 | 45 (17.6) |  | 25 (16.8) | 20 (18.7) |  | 1.08 (0.54-2.17) |
| Rheumatic disease: n (%) | 256 | 40 (15.6) |  | 21 (14.1) | 19 (17.8) |  | 1.33 (0.64-2.77) |
| Diabetes mellitus: n (%) | 256 | 62 (24.2) |  | 31 (20.8) | 31 (29.0) |  | 1.68 (0.91-3.12) |
| Active cancer/malignancy: n (%) | 251 | 54 (21.5) |  | 28 (19.2) | 26 (24.8) |  | 1.40 (0.74-2.67) |
| Previous bleeding: n (%) ^c^ | 249 | 13 (5.2) |  | 4 (2.7) | 9 (8.7) |  | 2.82 (0.76-10.40) |
| Creatinine clearance: n (%) | 256 |  |  |  |  |  |  |
| ≥60 *mL/min* |  | 103 (40.2) |  | 57 (38.3) | 46 (43.0) |  | Ref. |
| 30-59 *mL/min* |  | 60 (23.4) |  | 33 (22.1) | 27 (25.2) |  | 0.95 (0.48-1.88) |
| *<30 mL/min* |  | 15 (5.9) |  | 9 (6.0) | 6 (5.6) |  | 1.02 (0.32-3.18) |
| *Not tested* |  | 78 (30.5) |  | 50 (33.6) | 28 (26.2) |  | 0.63 (0.33-1.23) |
| Platelet count: n (%) | 256 |  |  |  |  |  |  |
| *≥100 x 10^9^/L* |  | 134 (52.3) |  | 73 (49.0) | 61 (57.0) |  | Ref. |
| *<100 x 10^9^/L* |  | 3 (1.2) |  | 0 (0) | 3 (2.8) |  | n/a |
| *Not tested* |  | 119 (46.5) |  | 76 (51.0 | 43 (40.2) |  | 0.59 (0.33-1.03) |
| VKA regimen: n (%) | 256 |  |  |  |  |  |  |
| *Acenocoumarol* |  | 203 (79.3) |  | 125 (83.9) | 78 (72.9) |  | Ref. |
| *Phenprocoumon* |  | 53 (20.7) |  | 24 (16.1) | 29 (27.1) |  | 1.57 (0.77-3.21) |
| VKA reversal agent used: n (%) | 256 | 58 (22.7) |  | 35 (23.5) | 23 (21.5) |  | 0.71 (0.37-1.39) |
| Antithrombotic agent use: n (%) | 256 | 19 (7.4) |  | 13 (8.7) | 6 (5.6) |  | 0.53 (0.18-1.56) |
| BMI: mean (SD) ^d^ | 244 | 27.7 (5.3) |  | 27.9 (5.6) | 27.4 (4.8) |  | 1.00 (0.95-1.05) |
| Smoking status: n(%) | 238 |  |  |  |  |  |  |
| *No* |  | 146 (61.3) |  | 91 (64.5) | 55 (56.7) |  | Ref. |
| *Yes* |  | 38 (16.0) |  | 22 (15.6) | 16 (16.5) |  | 1.17 (0.54-2.56) |
| *Quit* |  | 54 (22.7) |  | 28 (19.9) | 26 (26.8) |  | 1.65 (0.82-3.31) |
| Length of stay (days): median (IQR) ^d^ | 256 | 6 (3-10) |  | 5 (3-8.5) | 8 (4-12) |  | **1.07 (1.03-1.11)** |
| ICU/CCU stay during admission: n (%) | 256 | 40 (15.6) |  | 13 (8.7) | 27 (25.2) |  | **3.80 (1.80-8.05)** |
| CVC: n (%) | 256 | 13 (5.1) |  | 5 (3.4) | 8 (7.5) |  | 1.90 (0.56-6.40) |
| Spinal/epidural catheter: n (%) | 256 | 13 (5.1) |  | 7 (4.7) | 6 (5.6) |  | 1.38 (0.43-4.50) |
| **Surgery characteristics** |  |  |  |  |  |  |  |
| Non-elective: n (%) | 256 | 75 (29.3) |  | 41 (27.5) | 34 (31.8) |  | 1.23 (0.69-2.20) |
| Second surgery performed: n(%) | 256 | 19 (7.4) |  | 4 (2.7) | 15 (14.0) |  | **6.45 (1.96-21.21)** |
| Type of 1^st^ surgery: n (%) | 256 |  |  |  |  |  |  |
| *Urologic* |  | 40 (15.6) |  | 29 (19.5) | 11 (10.3) |  | Ref. |
| *Orthopaedic* |  | 89 (34.8) |  | 63 (42.3) | 26 (24.3) |  | 1.17 (0.48-2.84) |
| *Gastrointestinal* |  | 52 (20.3) |  | 24 (16.1) | 28 (26.2) |  | **3.83 (1.50-9.74)** |
| *Vascular* |  | 36 (14.1) |  | 16 (10.7) | 20 (18.7) |  | **3.74 (1.36-10.29)** |
| *Other* |  | 39 (15.2) |  | 17 (11.4) | 22 (20.6) |  | **3.06 (1.12-8.39)** |
| Spinal/epidural anaesthesia | 256 | 116 (45.3) |  | 75 (50.3) | 41 (38.3) |  | 0.62 (0.35-1.13) |
| Duration (minutes): median (IQR) ^d^ | 223 | 56 (33-92) |  | 54 (33-84) | 64 (32.5-109) |  | 1.00 (1.00-1.01) |
| Surgical bleeding risk | 256 |  |  |  |  |  |  |
| *High* |  | 209 (81.6) |  | 126 (84.6) | 83 (77.6) |  | Ref. |
| *Moderate* |  | 44 (17.2) |  | 21 (14.1) | 23(21.5) |  | 1.61 (0.79-3.26) |
| *Low* |  | 3 (1.2) |  | 2 (1.3) | 1 (0.9) |  | 1.13 (0.09-14.09) |
| **Care delivery characteristics** |  |  |  |  |  |  |  |
| DNR order: n (%) | 256 |  |  |  |  |  |  |
| *Resuscitate* |  | 185 (72.3) |  | 109 (73.2) | 76 (71.0) |  | Ref. |
| *Do not resuscitate* |  | 59 (23.0) |  | 33 (22.1) | 26 (24.3) |  | 1.17 (0.62-2.20) |
| *No order* |  | 12 (4.7) |  | 7 (4.7) | 5 (4.7) |  | 0.61 (0.17-2.19) |
| Weekend surgery: n (%) | 256 | 14 (5.5) |  | 10 (6.7) | 4 (3.7) |  | 0.54 (0.16-1.88) |
| Surgery outside regular hours: n (%) | 241 | 29 (12.0) |  | 17 (12.3) | 12 (11.7) |  | 1.05 (0.46-2.42) |
| Weekend admission: n (%) | 256 | 25 (9.8) |  | 12 (8.1) | 13 (12.1) |  | 1.43 (0.60-3.44) |
| Hospital type: n (%) | 256 |  |  |  |  |  |  |
| *General* |  | 137 (53.5) |  | 93 (62.4) | 44 (41.1) |  | Ref. |
| *Tertiary teaching* |  | 85 (33.2) |  | 44 (29.5) | 41 (38.3) |  | 1.92 (0.77-4.79) |
| *University medical centre* |  | 34 (13.3) |  | 12 (8.1) | 22 (20.6) |  | **3.94 (1.16-13.35)** |
| OR results in bold are significant (p <0.05)  All percentages are expressed as valid column percentages  AT9: **Antithrombotic Therapy and Prevention of Thrombosis, Ninth Edition guideline; CCU: cardiac care unit; CVC: central venous catheter;** DNR: do not resuscitate; hCVA: haemorrhagic cerebrovascular accident; ICU: intensive care unit; iCVA: ischaemic cerebrovascular accident; IQR: inter quartile range; n/a: coefficient could not be estimated; Ref: reference category; SD: standard deviation; SES: socioeconomic status; TIA: transient ischaemic attack; VKA: vitamin-K antagonist  ^a^ Adjusted for clustering at hospital level  ^b^ No AT9 risk classification is available for VKA indications other than atrial fibrillation, mechanical heart valves and venous thromboembolism  ^c^ Any previous bleeding event annotated in the medical record  ^d^ Analysed as continuous variable | | | | | | | |

| **Supplementary table 4** Demographic, clinical, surgical and care delivery characteristics by compliance status for low- and high thromboembolic risk patients. Additionally, univariable logistic regression results for under and overuse of bridging, adjusted for clustering on hospital level, are presented | | | | | | |  |
| --- | --- | --- | --- | --- | --- | --- | --- |
|  | Low Thromboembolic risk | | | High Thromboembolic risk | | |  |
|  | Compliant  N=89 | Overuse  N=46 | OR overuse reference: compliant  (95% CI)^a^ | Compliant  N=19 | Underuse  N=21 | OR underuse reference: compliant  (95% CI)^b^ |  |
| **Demographic characteristics** |  |  |  |  |  |  |  |
| Age: n (%) |  |  |  |  |  |  |  |
| *≥80 years* | 27 (30.3) | 16 (34.8) | Ref. | 10 (52.6) | 13 (61.9) | Ref. |  |
| 60-79 *years* | 56 (62.9) | 25 (54.3) | 0.90 (0.37-2.22) | 7 (36.8) | 8 (38.1) | 0.88 (0.23-3.40) |  |
| *<60 years* | 6 (6.7) | 5 (10.9) | 1.27 (0.24-6.70) | 2 (10.5) | 0 (0) | n/a |  |
| Female sex: n (%) | 34 (38.2) | 21 (45.7) | 1.56 (0.65-3.76) | 9 (47.4) | 8 (38.1) | 0.61 (0.16-2.35) |  |
| Lowest 25^th^ percentile SES: n (%) | 35 (40.2) | 13 (28.3) | 0.67 (0.27-1.67) | 6 (31.6) | 8 (38.1) | 1.32 (0.34-5.12) |  |
| **Clinical characteristics** |  |  |  |  |  |  |  |
| Atrial fibrillation: n (%) | 88 (98.9) | 43 (93.5) | 0.41 (0.02-7.85) | 16 (84.2) | 19 (90.5) | 1.79 (0.25-13.10) |  |
| Mechanical heart valve: n (%) | 0 (0) | 1 (2.2) | n/a | 5 (26.3) | 4 (19.0) | 0.60 (0.12-2.90) |  |
| Venous thromboembolism: n (%) | 3 (3.4) | 3 (6.5) | 2.39 (0.28-20.51) | 3 (15.8) | 1 (4.8) | 0.27 (0.02-3.09) |  |
| Previous thromboembolic event  during VKA interruption: n (%) | 0 (0) | 0 (0) | n/a | 1 (5.3) | 1 (4.8) | 0.93 (0.05-17.94) |  |
| iCVA/TIA: n (%) | 0 (0) | 0 (0) | n/a | 13 (68.4) | 16 (76.2) | 1.52 (0.353-6.50) |  |
| Thrombophilia: n (%) | 0 (0) | 0 (0) | n/a | 1 (5.3) | 0 | n/a |  |
| Coronary heart disease: n (%) | 26 (29.2) | 10 (21.7) | 0.66 (0.25-1.74) | 6 (31.6) | 8 (38.1) | 1.38 (0.34-5.55) |  |
| Heart failure: n (%) | 4 (4.5) | 4 (8.9) | 2.02 (0.39-10.57) | 1 (5.6) | 2 (10.0) | 1.90 (0.14-25.07) |  |
| Hypertension: n (%) | 39 (43.8) | 20 (43.5) | 1.39 (0.59-3.31) | 11 (57.9) | 16 (76.2) | 2.56 (0.60-10.88) |  |
| COPD: n (%) | 14 (15.7) | 7 (15.2) | 0.86 (0.27-2.69) | 5 (26.3) | 4 (19.0) | 0.67 (0.14-3.14) |  |
| Rheumatic disease: n (%) | 14 (15.7) | 9 (19.6) | 1.24 (0.39-3.92) | 3 (15.8) | 4 (19.0) | 1.23 (0.22-6.78) |  |
| Diabetes mellitus: n (%) | 12 (13.5) | 7 (15.2) | 1.32 (0.42-4.14) | 8 (42.1) | 4 (19.0) | 0.32 (0.07-1.42) |  |
| Active cancer/malignancy: n (%) | 18 (20.9) | 9 (20.0) | 0.60 (0.21-1.73) | 4 (21.1) | 5 (23.8) | 1.16 (0.25-5.44) |  |
| Previous bleeding: n (%)^c^ | 3 (3.4) | 5 (10.9) | 2.04 (0.37-11.21) | 1 (5.6) | 1 (5.0) | 0.88 (0.4-17.91) |  |
| VKA regimen: n (%) |  |  |  |  |  |  |  |
| *Acenocoumarol* | 73 (82.0) | 35 (76.1) | Ref. | 13 (68.4) | 17 (81.0) | Ref. |  |
| *Phenprocoumon* | 16 (18.0) | 11 (23.9) | 1.33 (0.43-4.09) | 6 (31.6) | 4 (19.0) | 0.51 (0.11-2.32) |  |
| VKA reversal agent used: n (%) | 17 (19.1) | 11 (23.9) | 1.40 (0.51-3.83) | 4 (21.1) | 9 (42.9) | 3.52 (0.76-16.38) |  |
| Antithrombotic agent use: n (%) | 6 (6.7) | 1 (2.2) | 0.23 (0.02-2.62) | 0 (0) | 1 (4.8) | n/a |  |
| BMI: mean (SD) ^d^ | 27.7 (5.5) | 26.4 (5.1) | 0.97 (0.89-1.06) | 28.2 (4.9) | 27.9 (5.6) | 0.99 (0.86-1.13) |  |
| Smoking status: n(%) |  |  |  |  |  |  |  |
| *No* | 58 (68.2) | 24 (58.5) | Ref. | 8 (47.1) | 9 (47.4) | Ref. |  |
| *Yes* | 15 (17.6) | 7 (17.1) | 1.04 (0.33-3.29) | 2 (11.8) | 3 (15.8) | 1.28 (0.15-11.06) |  |
| *Quit* | 12 (14.1) | 10 (24.4) | 1.74 (0.54-5.67) | 7 (41.2) | 7 (36.8) | 0.86 (0.19-3.93) |  |
| **Surgery characteristics** |  |  |  |  |  |  |  |
| Non-elective: n (%) | 17 (19.1) | 16 (34.8) | **2.72 (1.03-7.19)** | 7 (36.8) | 11 (52.4) | 1.89 (0.50-7.08) |  |
| Type of 1^st^ surgery: n (%) |  |  |  |  |  |  |  |
| *Urologic* | 23 (25.8) | 3 (6.5) | Ref. | 3 (15.8) | 2 (9.5) | Ref. |  |
| *Orthopaedic* | 38 (42.7) | 12 (26.1) | 3.62 (0.75-17.53) | 4 (21.1) | 13 (61.9) | 12.69 (0.70-231.02) |  |
| *Gastrointestinal* | 15 (16.9) | 13 (28.3) | **15.87 (3.02-83.42)** | 4 (21.1) | 2 (9.5) | 0.73 (0.3-16.90) |  |
| *Vascular* | 9 (10.1) | 7 (15.2) | **9.58 (1.49-61.42)** | 6 (31.6) | 0 (0) | n/a |  |
| *Other* | 4 (4.5) | 11 (23.9) | **27.43 (3.49-215.38)** | 2 (10.2) | 4 (19.0) | 4.19 (0.15-114.98) |  |
| Spinal/epidural anaesthesia | 51 (57.3) | 19 (41.3) | 0.56 (0.21-1.39) | 8 (42.1) | 8 (38.1) | 0.87 (0.23-3.26) |  |
| Duration (minutes): median (IQR) ^d^ | 55 (33-84) | 75 (38-126) | 1.01 (1.00-1.01) | 73 (44-131) | 51 (40-64) | 0.98 (0.96-1.00) |  |
| Surgical bleeding risk |  |  |  |  |  |  |  |
| *High* | 77 (86.5) | 34 (73.9) | Ref. | 17 (89.5) | 18 (85.7) | Ref. |  |
| *Moderate* | 11 (12.4) | 11 (23.9) | 2.77 (0.89-8.63) | 2 (10.5) | 3 (14.3) | 1.40 (0.19-10.18) |  |
| *Low* | 1 (1.1) | 1 (2.2) | 4.71 (0.21-107.16) | 0 (0) | 0 (0) | n/a |  |
| **Care delivery characteristics** |  |  |  |  |  |  |  |
| DNR order: n (%) |  |  |  |  |  |  |  |
| *Resuscitate* | 67 (75.3) | 35 (76.1) | Ref. | 13 (68.4) | 14 (66.7) | Ref. |  |
| *Do not resuscitate* | 20 (22.5) | 10 (21.7) | 1.05 (0.38-2.89) | 5 (26.3) | 7 (33.3) | 1.30 (0.31-5.38) |  |
| *No order* | 2 (2.2) | 1 (2.2) | 0.25 (0.02 -3.65) | 1 (5.3) | 0 (0) | n/a |  |
| Weekend surgery: n (%) | 5 (5.6) | 1 (2.2) | 0.37 (0.03-4.03) | 1 (5.3) | 3 (14.3) | 3.15 (0.27-36.85) |  |
| Surgery outside regular hours: n (%) | 8 (9.6) | 5 (11.6) | 1.64 (0.43-6.25) | 1 (5.3) | 4 (21.1) | 4.80 (0.45-51.64) |  |
| Weekend admission: n (%) | 5 (5.6) | 6 (13.0) | 1.38 (0.32-5.92) | 3 (15.8) | 3 (14.3) | 0.88 (0.15-5.33) |  |
| Hospital type: n (%) |  |  |  |  |  |  |  |
| *General* | 60 (67.4) | 17 (37.0) | Ref. | 11 (57.9) | 12 (57.1) | Ref. |  |
| *Tertiary teaching* | 23 (25.8) | 15 (32.6) | 1.90 (0.35-10.46) | 7 (36.8) | 6 (28.6) | 0.81 (0.18-3.77) |  |
| *University medical centre* | 6 (6.7) | 14 (30.4) | **9.01 (1.05-77.57)** | 1 (5.3) | 3 (14.3) | 2.83 (0.21-38.43) |  |
| OR results in bold are significant  All percentages are expressed as valid column percentages  **CCU: cardiac care unit; CVC: central venous catheter;** DNR: do not resuscitate; hCVA: haemorrhagic cerebrovascular accident; ICU: intensive care unit; iCVA: ischaemic cerebrovascular accident; IQR: inter quartile range; n/a: coefficient could not be estimated; Ref: reference category; SD: standard deviation; SES: socioeconomic status; TIA: transient ischaemic attack; VKA: vitamin-K antagonist  ^a^ Adjusted for clustering at hospital level  ^b^ Adjusting for clustering at hospital level was not possible due to small number of observations  ^c^ Any previous bleeding event annotated in the medical record  ^d^ Analysed as continuous variable | | | | | | | |
